# Associations of Face-to-Face and Instant Messaging Family Communication and Their Contents With Family Wellbeing and Personal Happiness Amidst the COVID-19 Pandemic

**DOI:** 10.3389/fpsyt.2022.780714

**Published:** 2022-03-29

**Authors:** Wei Jie Gong, Shirley Man Man Sit, Bonny Yee Man Wong, Socrates Yong Da Wu, Agnes Yuen Kwan Lai, Sai Yin Ho, Man Ping Wang, Tai Hing Lam

**Affiliations:** ^1^School of Public Health, The University of Hong Kong, Hong Kong, Hong Kong SAR, China; ^2^School of Nursing, The University of Hong Kong, Hong Kong, Hong Kong SAR, China; ^3^School of Nursing and Health Studies, Hong Kong Metropolitan University, Hong Kong, Hong Kong SAR, China

**Keywords:** communication contents, family wellbeing, happiness, instant messaging (IM), face-to-face (F2F)

## Abstract

**Background:**

Both face-to-face and instant messaging (IM) communication are important for families, but face-to-face communication has reduced amidst the COVID-19 pandemic. We examined the use and contents of both communication methods amidst the pandemic, their associations with family wellbeing and personal happiness, and the mediation effects of communication quality in Hong Kong Chinese adults.

**Methods:**

This population-based online survey enrolled 4,921 respondents in May 2020, who reported (i) any face-to-face or IM family communication when the pandemic was severe; (ii) communication contents being classified as neutral, positive, supportive, and negative; and (iii) communication quality, family wellbeing and personal happiness (score 0–10). Associations of family wellbeing and personal happiness with communication methods and contents (no communication excluded) were examined using linear regressions (β), adjusting for each other, sex, age, socioeconomic status, and the number of cohabitants. Mediating effects of communication quality on these associations were examined. Prevalence estimates were weighted by sex, age, and education of the general population. Interactions of methods and contents were examined.

**Results:**

Of 4,891 included respondents (female: 52.9%, 45–54 years: 37.7%, ≥65 years: 21.3%), 7.1% reported no communication, 12.7% face-to-face communication only, 26.7% IM only, and 53.4% both methods. More males and those at younger ages, had lower socioeconomic status, or fewer cohabitants showed no family communication or face-to-face only. More respondents reported neutral (83.1–99.3%) than positive (42.1–62.2%), supportive (37.5–54.8%), and negative (10.9–34.5%) contents despite communication methods. Communication quality was higher with both methods than IM only, face-to-face only, and no communication (scores: 6.7 vs. 4.5–6.6, all *P* ≤ 0.02). Better family wellbeing and personal happiness were associated with using IM only (adjusted βs: 0.37 and 0.48) and both methods (0.37 and 0.42) than face-to-face only, and positive (0.62 and 0.74) or supportive (0.45 and 0.46) contents (all *P* ≤ 0.001). Communication quality mediated 35.2–93.5% of these associations. Stronger associations between positive contents and family wellbeing showed in both methods and face-to-face only than IM only (*P* for interaction = 0.006).

**Conclusions:**

We have first shown that, amidst the COVID-19 pandemic, family IM communication and positive and supportive contents may promote family wellbeing and personal happiness. People with no family communication may need assistance.

## Introduction

The novel coronavirus (COVID-19) pandemic poses global threats to the wellbeing of families and individuals. Increased family-related mental burdens and personal unhappiness have been reported amidst social disruptions and financial insecurity (1–3). Health, social, and economic challenges and adverse consequences from the pandemic are increasing. Posited by Prime and Wade's framework, individuals and families would be differentially influenced by the pandemic and well-functioning families, consisting of effective communication, organization, and belief systems, would be more resilient to these risks (4).

Family communication is a core process of the concepts of family wellbeing and family system and is foundational in maintaining family relationships and fostering individual wellbeing, as stressed in many Western theories and in Chinese culture (4–7). The pandemic triggers various stressors and intensifies the needs of feeling safe, hopeful, and socially connected (8). It has dramatically changed the habitual way many families interact and communicate, especially among those who were separated across different households amidst lockdowns and other social distancing restrictions (9, 10). With such challenges disrupting usual face-to-face communication, communication via digital tools such as instant messaging (IM) has increased substantially (11). Further understanding of how and what families are communicating amidst the pandemic is needed in preparation of the “new normal” and future pandemics.

Previous studies mostly focused on IM communication in social networks in romantic relationships, friendship, and working relationships, but rarely within the whole family (12). IM users did not have emotionally closer feelings with network members when offline (13), and longer duration of IM interactions did not predict better subjective happiness (14). We searched PubMed and Web of Science using a combination of keywords including “COVID-19”, “coronavirus”, “family”, “communication”, “face-to-face”, “instant messaging” and “wellbeing” up to 21 August 2021 to identify IM use in closed communication circles such as families. Only four of our previous papers separately reported the use of family IM chat groups associated with higher family functioning and wellbeing (15) and with personal happiness amidst the pandemic (16), sharing of family life information associated with higher family wellbeing (17), and socioeconomic disparities in using different digital communication methods for family communication (18). We found no reports on the contents of family communication or their associations with family and personal wellbeing amidst the pandemic.

Throughout the life course, family communication is crucial for a balanced family system and involves the exchange and sharing of information, knowledge, values, and beliefs (6, 19). The content of family communication and type of information being shared can shape behaviors, emotions, and sense of self (20, 21), and may thus influence family and individual wellbeing. In addition to providing support against social isolation and loneliness during the pandemic, family communication can also be an important source of health information (22, 23). Hong Kong has had no lockdowns during the pandemic, but almost 100% voluntary mask-wearing in the public started within a few weeks of the first COVID-19 outbreak. The government implemented social distancing regulations since 29 March 2020 (24), which deterred family gatherings and face-to-face interaction with family members. With such disruptions to in-person communication, communication via IM platforms such as WhatsApp and WeChat became popular alternatives, allowing for convenient and instant exchange of texts, voice messages, images including pictures and photos, videos, and audio clips. The pandemic has led to more older adults making use of the internet and smartphones to stay connected to family members (25, 26). In Hong Kong, both face-to-face communication and IM were previously found to be common methods of family communication (18).

We hypothesized that, (i) the use of face-to-face and IM family communication and their corresponding contents amidst the COVID-19 pandemic are associated with family and personal wellbeing, (ii) these associations are mediated by family communication quality, and (iii) communication methods moderate the associations between communication contents and outcomes. This study aimed to examine the use and contents of face-to-face and IM family communication amidst the COVID-19 pandemic, their associations with family wellbeing and personal happiness, the mediating effects of communication quality, and whether the moderating effects of communication methods exist.

## Materials and Methods

### Study Design and Participants

The Hong Kong Jockey Club SMART Family-Link project (2018–2022) is a large cross-sectoral collaboration between The University of Hong Kong and 26 local family service providers, aiming to advance information and communication technology (ICT) use in family services and to promote family wellbeing and happiness in local people (27). Under this project, the Family amidst COVID-19 survey (FamCov1) was designed to examine ICT related behaviors, attitudes and concerns toward the COVID-19 pandemic, and personal and family wellbeing in Hong Kong families. It was a population-based, cross-sectional survey conducted during 26–31 May 2020 to recruit a sample as large as possible within 6 days when the second wave of COVID-9 outbreak was under control. The target population was Chinese adults in Hong Kong aged 18 years or above who can read and write in Traditional Chinese. Those who were psychologically or physically unable to complete the whole online questionnaires were excluded. Email invitations to join the online survey were sent to both probability and non-probability-based online panels of the Hong Kong Public Opinion Research Institute, a well-known local survey agency (28). Respondents voluntarily answered the questionnaires with no incentive. Among the 20,103 opened invitation emails, 4,921 (24.5%) respondents completed the whole survey. After excluding 30 respondents that had no family members, 4,891 respondents (99.4%) were included in this study.

Details of the methods have been reported in three of our papers using the same data (3, 16, 29), showing that the perceived benefits and harms of COVID-19 were associated with sociodemographic factors (3), the fear of COVID-19 showed socioeconomic differences and was associated with perceived benefits and harms of COVID-19 (29), and that the use of different IM functions in family e-chat groups amidst the pandemic was associated with family wellbeing and personal happiness (16).

Ethics approval was obtained from the Institutional Review Board of the University of Hong Kong/Hospital Authority Hong Kong West Cluster (Reference Number: UW 20-238). All respondents gave informed consent before starting the survey. This study was carried out in accordance with the Declaration of Helsinki and all its amendments.

### Measurements

#### Independent Variables

The definitions of family (“family members who are related through biological, marital, cohabitation, or/and emotional bonding”) and IM e-chat group (“a group of 3 or more people in IM communication applications such as WhatsApp or WeChat, etc.”) were given before related questions. Face-to-face communication with family was asked by the question, “When the pandemic was severe, on average per week, how many days did you communicate face-to-face with family members?”. Respondents answering 0 days or 1–7 days were regarded as either having none or had face-to-face communication with family, respectively. IM communication was asked by two questions, “When the pandemic was severe, on average per week, how many days did you receive/send instant messages from/to family members in family e-chat groups?”. Respondents answering 0 days for both questions or 1–7 days for one/both questions were regarded as having none or had IM communication with family, respectively.

The corresponding contents of face-to-face and IM were asked using multiple-choice questions, “When the pandemic was severe, what contents did you communicate face-to-face with family members?” or “When the pandemic was severe, what contents in the instant messages you receive or send from/to family members in family e-chat groups?”. The answers include COVID-19-related information, self/family-related things in daily life, self/family-related and unrelated happy/funny things, and related unhappy things, showing care, encouragement, appreciation, good wishes, other health information, and others (e.g., daily life information, news, and current affairs, etc.,).

#### Dependent Variables

Family communication quality was measured using a single item, “How do you find the quality of communication between you and your family members?”, which has been used in our previous study (30). Family health, harmony, and happiness (3Hs) were measured using the Family Wellbeing Scale, with validity shown in our previous studies (7, 31). Family wellbeing was calculated as the average score of family 3Hs. Personal happiness was examined using a single item, “How happy do you think you are, “with reliability and validity shown in previous surveys (32). All outcomes were measured on an 11-point scale (score 0–10), with higher scores indicating better outcomes, which allows more differentiation of the answers than Likert scales with fewer options (33).

#### Covariates

Information on sociodemographic characteristics was collected, including sex, age group (18–24 years, 25–34 years, 45–64 years, and 65 years or above), education (primary or below, secondary, post-secondary, and university or above), monthly household income (no income, less than HK$ 4,000, HK$ 4,000–9,999, HK$ 10,000–19,999, HK$ 20,000–29,999, HK$ 30,000–39,999, and HK$ 40,000 or higher) (US$ 1.0 = HK$ 7.8), housing type (rented and owned), and household size (number of cohabitants, including the respondent).

### Statistical Analysis

Education was dichotomized as secondary or below and tertiary. Monthly household income per person (income being divided by household size) was dichotomized as lower and higher using the median household income and household size of the 2019 Hong Kong census data (34). Socioeconomic status was calculated as a composite score of education (0 = secondary or below, 1 = tertiary), income (0 = lower, 1 = higher), and housing (0 = rented, 1 = owned) and analyzed as low (0–1), medium (2), and high (3). Communication methods were divided into four groups, including no communication, face-to-face only, IM only, and both methods. Contents in family communication were divided into four groups by their affective interpretation, including neutral (self/family-related things in daily life, COVID-19-related information, other health information, and others, e.g., daily life information, news, and current affairs, etc.,), positive (self/family-related and unrelated happy/funny things), supportive (showing care, encouragements, appreciations, and good wishes) and negative (self/family-related unhappy things) contents. Among them, neutral, positive, and negative contents have been used before (35, 36). We especially distinguished supportive from positive contents, because Chinese people tend to have implicit and indirect expressions instead of direct verbal expressions of supportive contents (37–39).

The raw data and prevalence estimates were weighted by sex, age, and educational attainment of the 2019 Hong Kong census data (40, 41). Pairwise comparisons using Chi-square tests for categorical variables and *t*-tests for continuous variables were used to compare the characteristics and outcomes of respondents having no family communication, face-to-face only, and IM only with those using both methods, and to compare the contents in the following 3 pairs of communication methods: face-to-face only vs. IM only, both methods vs. face-to-face only, and both methods vs. IM only, with Bonferroni adjusted level of significance (0.05/3 = 0.017).

Adjusted regression coefficients (βs) and their 95% confidence intervals (CIs) were estimated using multivariable linear regressions to estimate the associations of outcomes, including family wellbeing and personal happiness, with the four communication methods in Model I, with the four kinds of contents in Model II, and with both methods and contents in Model III, adjusted for sex, age groups, socioeconomic status, and the number of cohabitants. People having no family communication were excluded in Model II and Model III. Based on model III, we additionally examined the mediating effects of family communication quality on these associations using the Baron and Kenny approach (42), and whether the mediating (indirect) effects were significant were examined using the Sobel tests. The bias-corrected bootstrap CIs of the total, indirect and direct effects were calculated with 1,000 replications, adjusted for sex, age group, socioeconomic status, and the number of cohabitants. The moderating effects of communication methods on the associations of contents with outcomes were examined by additionally including the interaction terms of methods and contents in corresponding regression models. A 2-sided *P* < 0.05 was considered statistically significant. All statistical analyses were performed using STATA version 15.0 (StataCorp LP, College Station, TX, USA).

## Results

Of the 4,891 respondents included in this study, after weighting, 52.9% of them were female, with the mean age of 43.5 years (37.7% aged 45–64 years and 21.3% aged ≥65 years). Details of their sociodemographic characteristics have been previously reported (3, 29). Table 1 shows that after weighting, over half of respondents (53.4%) communicated with family members using both methods (face-to-face and IM messages), followed by IM only (26.7%), face-to-face only (12.7%), and no family communication (7.1%). Compared with those using both methods, more respondents having no family communication or having face-to-face communication only were male (52.5 and 65.1%, respectively, vs. 46.2%), at younger ages, and had lower socioeconomic status (low: 61.6 and 52.8%, respectively, vs. 48.7%) and fewer cohabitants (all *P* ≤ 0.03); while more of those using IM only were female (61.2 vs. 53.8%), at older ages, and had lower socioeconomic status (low: 56.6 vs. 48.7%) and fewer cohabitants (all *P* ≤ 0.001). The unweighted characteristics are shown in Supplementary Table 1.

**Table 1:** Weighted characteristics and family and personal outcomes by communication methods^a^.

**Characteristics**	**No family communication (*****n*** **=** **348, 7.1%)**		**Face-to-face only (*****n*** **=** **619, 12.7%)**		**IM only (*****n*** **=** **1,304, 26.7%)**		**Both methods (*n* = 2,607, 53.4%)**	**Total**
	***n* (%)**	***P* (vs. both methods)^**c**^**	***n* (%)**	***P* (vs. both methods)^**c**^**	***n* (%)**	***P* (vs. both methods)^**c**^**	***n* (%)**	***n* (%)**
Sex		0.03		<0.001		<0.001		
Male	183 (52.5)		403 (65.1)		505 (38.8)		1,204 (46.2)	2,295 (47.1)
Female	165 (47.5)		216 (34.9)		799 (61.2)		1,403 (53.8)	2,583 (52.9)
Age group (years)		0.03		<0.001		<0.001	
18–24	43 (12.4)		83 (13.3)		21 (1.6)		270 (10.4)	416 (8.5)
25–44	127 (36.6)		244 (39.4)		374 (28.7)		837 (32.1)	1,581 (32.4)
45–64	127 (36.6)		200 (32.3)		545 (41.8)		967 (37.1)	1,839 (37.7)
≥65	50 (14.4)		93 (15.0)		365 (28.0)		533 (20.5)	1,041 (21.3)
Education		0.002		0.07		<0.001		
Secondary or below	248 (72.3)		370 (60.1)		904 (70.2)		1,661 (64.0)	3,183 (65.7)
Tertiary or above	95 (27.7)		246 (39.9)		385 (29.9)		936 (36.0)	1,662 (34.3)
Monthly household income per person		0.01		0.33		<0.001	
Lower	180 (58.3)		274 (52.1)		650 (56.8)		1,098 (49.7)	2,201 (52.6)
Higher	129 (41.7)		252 (47.9)		495 (43.2)		1,111 (50.3)	1,986 (34.3)
Housing type		<0.001		<0.001		0.06		
Rented	167 (49.3)		273 (45.8)		463 (36.0)		842 (33.0)	1,744 (36.6)
Owned	172 (50.8)		322 (54.2)		822 (64.0)		1,709 (67.0)	3,025 (63.4)
Socioeconomic status^b^		<0.001		<0.001		<0.001		
Low	187 (61.6)		267 (52.8)		644 (56.6)		1,063 (48.7)	2,160 (52.3)
Medium	80 (26.5)		164 (32.5)		363 (31.9)		768 (35.2)	1,376 (33.3)
High	36 (11.9)		75 (14.8)		131 (11.5)		353 (16.2)	595 (14.4)
Number of cohabitants		<0.001		<0.001		<0.001		
Mean ± SD	1.9 ± 1.4		2.3 ± 1.1		1.8 ± 1.1		2.5 ± 1.1	2.3 ± 1.3
Family and personal outcomes, mean ± SD^d^								
Family communication quality	4.5 ± 2.8	<0.001	6.0 ± 2.2	<0.001	6.6 ± 1.9	0.02	6.7 ± 1.8	6.6 ± 2.4
Family wellbeing	5.6 ± 2.3	<0.001	6.6 ± 1.9	<0.001	7.1 ± 1.6	0.003	7.3 ± 1.5	7.1 ± 1.6
Personal happiness	5.0 ± 2.4	<0.001	5.3 ± 2.2	<0.001	6.1 ± 2.1	0.43	6.2 ± 2.0	6.0 ± 2.1

*IM, Instant messaging; SD, Standard deviation*.

a *Weighted by sex, age, and education of the 2019 Hong Kong census data. Respondents with missing data were excluded. Total percentages may not be 100.0% after rounding. Frequencies may not add up tp the total numbers after weighting*.

b *Socioeconomic status: a composite score of education (0 = secondary or below, 1 = tertiary), income (0 = lower, 1 = higher), and housing (0 = rented, 1 = owned), analyzed as low (0–1), medium (2) and high (3)*.

c *Pairwise comparisons using Chi-square test for categorical variables and t-test for continuous variables with Bonferroni adjusted level of significance: 0.05/3 = 0.017*.

d *Score 0–10, higher scores indicate better outcomes*.

Table 2 shows that after weighting, in face-to-face communication, self/family-related things in daily life (79.5%) were the most frequent contents, followed by information of COVID-19 (78.2%), self/family-related happy/funny things (47.1%), others (e.g., daily life information, news, and current affairs, etc.,) (46.0%), showing care (42.8%), other health information (30.8%), self/family-related unhappy things (29.3%), self/family-unrelated happy/funny things (26.9%), encouragements (14.9%), appreciations (12.1%), and good wishes (11.9%). In IM messages, information of COVID-19 (80.6%) was the most common contents, followed by self/family-related things in daily life (59.6%), showing care (41.2%), and others (40.2%), whereas encouragements (16.9%), good wishes (16.3%), self/family-related unhappy things (16.0%), and appreciations (8.7%) were the least 4 common ones. When contents were grouped into 4 kinds, fewer respondents reported neutral (88.1 vs. 98.4%), positive (43.9 vs. 60.8%), and negative contents (16.0 vs. 29.3%) in IM communication than in face-to-face communication (all *P* <0.001). The unweighted percentages of contents are shown in Supplementary Table 2.

**Table 2:** Weighted percentages of contents by communication methods^a^.

**Contents**	**Total**			**One method only**			**Both methods (*****n*** **=** **2,607)**		
	**F2F, *n* (%) (*n* = 3,225)**	**IM, *n* (%) (*n* = 3,911)**	***P* ^b^**	**F2F only, *n* (%) (*n* = 619)**	**IM only, *n* (%) (*n* = 1,304)**	***P* ^c^**	***n* (%)**	***P*^c^ (vs. F2F only**	***P*^c^ (vs. IM only)**
Self/family-related things in daily life	2,564 (79.5)	2,330 (59.6)	<0.001	454 (73.3)	675 (51.7)	<0.001	2,233 (85.7)	<0.001	<0.001
Information of COVID-19	2,522 (78.2)	3,150 (80.6)	0.02	461 (74.4)	1,065 (81.7)	<0.001	2,271 (87.2)	<0.001	<0.001
Self/family-related happy/funny things	1,520 (47.1)	1,481 (37.9)	<0.001	219 (35.4)	439 (33.7)	0.46	1,495 (57.4)	<0.001	<0.001
Others (e.g., daily life information, news, and current affairs, etc.)	1,483 (46.0)	1,572 (40.2)	<0.001	241 (39.0)	465 (35.7)	0.16	1,468 (56.3)	<0.001	<0.001
Showing care	1,380 (42.8)	1,611 (41.2)	0.17	226 (36.5)	613 (47.0)	<0.001	1,347 (51.7)	<0.001	0.01
Other health information	994 (30.8)	1,384 (35.4)	<0.001	181 (29.3)	495 (38.0)	<0.001	1,093 (41.9)	<0.001	0.02
Self/family-related unhappy things	945 (29.3)	627 (16.0)	<0.001	120 (19.4)	142 (10.9)	<0.001	899 (34.5)	<0.001	<0.001
Self/family-unrelated happy/funny things	866 (26.9)	756 (19.3)	<0.001	123 (19.8)	219 (16.8)	0.10	881 (33.8)	<0.001	<0.001
Encouragements	479 (14.9)	661 (16.9)	0.02	44 (7.2)	239 (18.3)	<0.001	581 (22.3)	<0.001	0.004
Appreciations	390 (12.1)	341 (8.7)	<0.001	46 (7.4)	122 (9.4)	0.16	414 (15.9)	<0.001	<0.001
Good wishes	382 (11.9)	636 (16.3)	<0.001	37 (5.9)	262 (20.1)	<0.001	492 (18.9)	<0.001	0.36
Different kinds of contents^d^									
Neutral contents	3,174 (98.4)	3,444 (88.1)	<0.001	602 (97.3)	1,083 (83.1)	<0.001	2,589 (99.3)	<0.001	<0.001
Positive contents	1,694 (60.8)	1,717 (43.9)	<0.001	262 (42.3)	550 (42.1)	0.95	1,621 (62.2)	<0.001	<0.001
Supportive contents	1,465 (45.4)	1,796 (45.9)	0.68	232 (37.5)	701 (53.7)	<0.001	1,428 (54.8)	<0.001	0.54
Negative contents	945 (29.3)	627 (16.0)	<0.001	120 (19.4)	142 (10.9)	<0.001	899 (34.5)	<0.001	<0.001

*F2F, Face-to-face; IM, Instant messaging*.

a *Weighted by sex, age, and education of the 2019 Hong Kong census data. Respondents with no family communication and those with missing data were excluded. Contents were ranked by their weighted percentages in total face-to-face*.

b *Chi-square test*.

c *Pairwise comparisons using Chi-square test and Bonferroni adjusted level of significance: 0.05/3 = 0.017*.

d *Neutral contents: self/family-related things in daily life, information of COVID-19, other health information, and others (e.g., daily life information, news, and current affairs, etc.,). Positive contents: self/family-related and unrelated happy/funny things. Supportive contents: showing care, encouragements, appreciations, and good wishes. Negative contents: self/family-related unhappy things*.

Compared with face-to-face communication only, IM only included less self/family-related things in daily life (51.7 vs. 73.3%) and self/family-related unhappy things (10.9 vs. 19.4%), but more information of COVID-19 (81.7 vs. 74.4%), showing care (47.0 vs. 36.5%), other health information (38.0 vs. 29.3%), encouragements (18.3 vs. 7.2%), and good wishes (20.1 vs. 5.9%) (all *P* < 0.001). For the 4 kinds of contents, it included less neutral (83.1 vs. 97.3%) and negative contents (10.9 vs. 19.4%) but more supportive contents (53.7 vs. 37.5%) (all *P* < 0.001). Using both methods included higher percentages of almost all contents than using one method only (all *P* ≤ 0.02) except good wishes compared with IM only (18.9 vs. 20.1%, *P* = 0.36). In general, using both methods contained more neutral, positive, and negative contents than using one method only (*P* < 0.001), except supportive contents (both 54.8% vs. IM only 53.7%) (*P* = 0.54).

Table 3 shows that after excluding those with no communication, when communication methods and contents were included in the same models, compared with using face-to-face communication only, using IM only and using both methods were associated with higher levels of family communication quality, family wellbeing, and personal happiness (Model III, adjusted βs: 0.30–0.48, all *P* < 0.001). Only positive, supportive and negative contents were associated with higher levels of family communication quality (adjusted βs: 0.27–0.70, all *P* < 0.001), and only positive and supportive contents were associated with higher levels of family wellbeing and personal happiness (adjusted βs: 0.45–0.74, all *P* < 0.001).

**Table 3:** Associations of communication methods with outcomes, adjusted β (95% CIs) (*n* = 4,891).

**Family communication**	**Family communication quality^**a**^**	**Family wellbeing^**a**^**	**Personal happiness^**a**^**
Model I^b^ (*n* = 4,891)			
Methods			
Face-to-face only	0	0	0
No communication	−1.67 (−1.94, −1.39)^***^	−1.08 (−1.31, −0.85)^***^	−0.33 (−0.62, −0.04)^*^
IM only	0.35 (0.15, 0.55)^**^	0.40 (0.23, 0.57)^***^	0.53 (0.32, 0.74)^***^
Both methods	0.58 (0.40, 0.75)^***^	0.57 (0.43, 0.72)^***^	0.63 (0.45, 0.82)^***^
Model lI^c^ (*n* = 4,571, no communication excluded)			
Kinds of contents			
Neutral			
Yes (vs. No)	0.39 (0.11, 0.67)^**^	0.20 (−0.03, 0.44)	0.25 (−0.05, 0.55)
Positive			
Yes (vs. No)	1.00 (0.89, 1.11)^***^	0.48 (0.68, 0.87)^***^	0.81 (0.69, 0.94)^***^
Supportive			
Yes (vs. No)	0.90 (0.79, 1.02)^***^	0.67 (0.57, 0.76)^***^	0.66 (0.53, 0.78)^***^
Negative			
Yes (vs. No)	0.82 (0.69, 0.95)^***^	0.51 (0.40, 0.62)^***^	0.37 (0.23, 0.51)^***^
Model III ^c^ (*n* = 4,571, no communication excluded)			
Methods			
Face-to-face only	0	0	0
IM only	0.35 (0.16, 0.53)^***^	0.37 (0.21, 0.53)^***^	0.48 (0.28, 0.69)^***^
Both methods	0.30 (0.14, 0.46)^***^	0.37 (0.23, 0.51)^***^	0.42 (0.24, 0.60)^***^
Kinds of contents			
Neutral			
Yes (vs. No)	0.21 (−0.06, 0.49)	0.08 (−0.16, 0.32)	0.20 (−0.11, 0.50)
Positive			
Yes (vs. No)	0.70 (0.58, 0.83)^***^	0.62 (0.51, 0.73)^***^	0.74 (0.59, 0.88)^***^
Supportive			
Yes (vs. No)	0.62 (0.51, 0.74)^***^	0.45 (0.35, 0.56)^***^	0.46 (0.33, 0.59)^***^
Negative			
Yes (vs. No)	0.27 (0.13, 0.41)^***^	0.05 (−0.07, 0.17)	0.15 (−0.30, 0.01)

*CI, Confidence interval; IM, Instant messaging*.

* *P < 0.05*.

** *P < 0.01*.

*** *P < 0.001*.

a *Score 0–10, higher scores indicate better outcomes, β is the score versus that for face-to-face only as reference*.

b *Model I and II: adjusted for sex, age group, socioeconomic status, and number of cohabitants*.

c *Model III: including communication methods and contents in one model, adjusted for sex, age group, socioeconomic status, number of cohabitants, and mutually adjusted for each other*.

Family communication quality partially mediated the associations of communication methods and positive contents with family wellbeing (proportion mediated: 50.1–76.1%) and personal happiness (proportion mediated: 35.2–51.4%), and the associations of supportive contents with personal happiness (73.9%), and almost fully mediated the association of supportive contents with family wellbeing (93.5%, *P* for direct effect = 0.35) (Table 4).

**Table 4:** Adjusted total, indirect, and direct effects of communication methods and positive and supportive contents on outcomes mediated by family communication quality, adjusted β (95% CIs) (*n* = 4,571)^a^.

**Mediation**	**Family wellbeing^**b**^**	**Personal happiness^**b**^**
IM only vs. Face-to-face only		
Relative total effect	0.37 (0.21, 0.53)^***^	0.48 (0.27, 0.68)^***^
Relative indirect effect	0.23 (0.09, 0.38)^***^	0.19 (0.07, 0.30)^***^
Relative direct effect	0.14 (0.04, 0.23)^**^	0.29 (0.11, 0.47)^**^
Proportion of relative total effect mediated	63.0%	39.4%
Both vs. Face-to-face only		
Relative total effect	0.38 (0.25, 0.52)^***^	0.44 (0.26, 0.62)^***^
Relative indirect effect	0.19 (0.07, 0.32)^***^	0.16 (0.06, 0.25)^***^
Relative direct effect	0.19 (0.11, 0.28)^***^	0.29 (0.13, 0.44)^***^
Proportion of relative total effect mediated	50.1%	35.2%
Positive contents (Yes vs. No)		
Total effect	0.62 (0.51, 0.73)^***^	0.74 (0.59, 0.88)^***^
Indirect effect	0.47 (0.38, 0.56)^***^	0.38 (0.30, 0.45)^***^
Direct effect	0.15 (0.08, 0.21)^***^	0.36 (0.23, 0.48)^***^
Proportion of total effect mediated	76.1%	51.4%
Supportive contents (Yes vs. No)		
Total effect	0.46 (0.35, 0.55)^***^	0.46 (0.33, 0.59)^***^
Indirect effect	0.43 (0.35, 0.51)^***^	0.34 (0.27, 0.40)^***^
Direct effect	0.03 (−0.03, 0.09)	0.12 (0.01, 0.24)^*^
Proportion of total effect mediated	93.5%	73.9%

*CI, Confidence interval; IM, Instant messaging*.

* *P < 0.05*.

*** *P < 0.01*.

*** *P < 0.001*.

a *Respondents having no family communication were excluded. Results were adjusted for sex, age group, socioeconomic status, and number of cohabitants, neutral contents, negative contents, and mutually adjusted for each other*.

b *Score 0–10, higher scores indicate better outcomes*.

**Figure 1:**
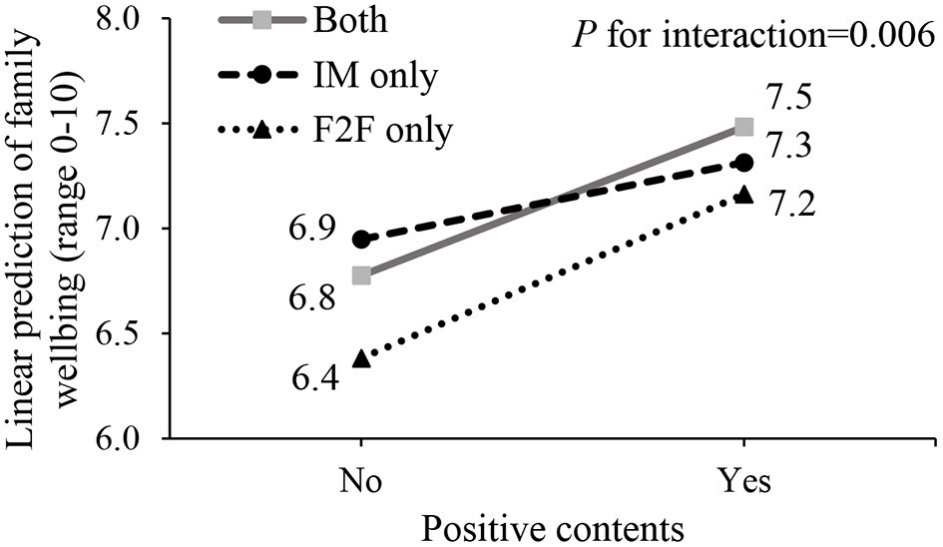
The linear prediction of family wellbeing by communication methods and positive contents^a^. ^a^IM, Instant messaging; F2F, Face-to-face.

Communication methods moderated the associations of positive contents with family wellbeing (*P* for interaction=0.006) (Figure 1). Positive contents had stronger associations with better family wellbeing in using both methods (estimated score changes: 0.71, 95% CI: 0.57–0.85) and face-to-face only (0.78, 95% CI: 0.53–1.03) than in IM only (0.37, 95% CI: 0.18–0.56) (*P* = 0.004 and 0.009, respectively).

## Discussion

This is the first study to report family communication methods and contents were independently associated with family communication quality, family wellbeing, and personal happiness, showing that better family wellbeing and personal happiness were associated with using IM only and both methods than face-to-face communication only, and were associated with having positive and supportive contents in family communication. About half to almost all these associations were mediated by communication quality. Communication methods moderated the association between positive contents and family wellbeing, showing stronger associations of family wellbeing with both methods and face-to-face only than IM only. These results are consistent with our three hypotheses.

Our results highlight the importance of using IM messages to communicate with family members amidst the COVID-19 pandemic, which showed better outcomes than face-to-face communication only. Due to physical isolation and social distancing, traditional face-to-face family communication and usual family gatherings have been disrupted, while digital communication via IM tools is increasingly used and often the only option in maintaining familial interactions in separated households (43). Those who could only use IM communication may value the interaction more and thus feel connected and supported. More men and younger people used face-to-face communication only probably because some might choose not to or seldom use IM messages for family communication even under social distancing restrictions. Consistent with previous studies, women and older people cared more about family affairs and participated in more IM family communication (17, 44). Their behaviors may be encouraged by perceived usefulness and enjoyment, attachment motivation, and relationship commitment, which were predictors of the intention of continuously using IM to sustain interpersonal relationships (45).

We found that when using both IM and face-to-face communication within the family context, almost all contents increased to a greater or lesser degree (15.9–87.2%) than using one method only (face-to-face only: 5.9–74.4%, IM only: 9.4–81.7%), except good wishes which were similar to IM only. Using both methods means more communication. IM messages may act as a supplement of face-to-face communication as an additional way of conversing even when face-to-face is possible, as IM is more convenient. For example, more COVID-19 and health-related information can be promptly shared in their original formats via IM messages, such as long texts, web links, photos, and short videos. Also, as Chinese people tend to indirectly express their encouragements, appreciations, and good wishes to family members (37–39), IM has become a brand-new platform to deliver and convey supportive contents beyond merely text, with emojis and many readily available e-messages for more vivid and intimate expressions (46). Face-to-face communication contains more self/family-related things in daily life and unhappy things and can provide better communication satisfaction through verbal, facial, and body language but is restricted by physical location (47).

All kinds of contents were associated with better family communication quality when sociodemographic characteristics were controlled (Model II), but only positive and supportive contents remained associated with family wellbeing and personal happiness when communication methods were also controlled (Model III). In a well-functioning family, members should be willing to share all kinds of contents, including negative ones. An effective coping mechanism in combating stress of negative life events is to seek comfort and help within social support networks such as family (48), through the sharing of affection and empathy, and giving encouragement, advice, and practical help such as health information (49). COVID-19 and health-related information have been widely spread amidst the pandemic, and the related sharing and forwarding behaviors could mean both showing care to family members and a cause of the ongoing infodemic, pandemic fear, and mental health burdens (50, 51). Previous studies have linked positive and supportive contents with confidence and competence among family members, while negative contents such as criticism were associated with lower self-esteem and defiance (20, 52). While open and direct expression of affection, both verbal and non-verbal, are encouraged in Western families (53), such as saying “I love you”, this is not common in Chinese households, where strong emotions are typically held back, stemming from a historical emphasis on the regulation of social behaviors and expression of emotions (54, 55). Such differences can also be observed in the discussion of funny and humorous topics within families (56). Considering the relatively low weighted percentages of positive and supportive contents (positive: 42.1–62.2%; supportive: 37.5–54.8% vs. neutral: 83.1–99.3%), increasing these contents through IM first may lead to increased use in face-to-face communication, which may promote family wellbeing and personal happiness. Intervention studies on IM use to deliver such contents to promote family and individual wellbeing are warranted.

The moderating effect of communication methods showed that positive contents in face-to-face communication only and both methods were more strongly associated with better family wellbeing than in IM messages, suggesting that sharing self/family-related and unrelated happy/funny things by IM only may be less effective for maintaining and nourishing family relationships. In face-to-face communication, non-verbal language, such as laughter and smiles, can give real-time positive feedbacks and immediately create a happy and enjoyable atmosphere (57). According to the attachment theory, pleasant and frequent interactions with others contribute to individual mental and emotional wellbeing (45), which may evoke better family wellbeing in family communication.

The mediation effects of family communication quality can provide new evidence to Prime and Wade's framework (4). Amidst the COVID-19 pandemic, family communication provides clear information, emotional sharing, collaborative problem-solving, and dyadic and family coping to connect family members and share beliefs. Quality communication, such as using both face-to-face and IM communication and including positive or supportive contents, can thus provide security and hope for vulnerable members during periods of stress (4), shown as higher perceived family wellbeing and personal happiness in the present study.

In Hong Kong, the most westernized city in China, the high penetration rate of smartphones (91.5% in 2019) and the Internet (87.0% in 2019) means most people can conveniently use social media and IM messages (58, 59). With 93.6% of the population being Chinese, Hong Kong people highly value family relationships, which are influenced by collectivism and Confucius ideals in traditional Chinese culture (31). However, we found that 7.1% of people had no family communication and they reported the lowest family communication quality, family wellbeing, and personal happiness. They tended to be in low socioeconomic status and could be vulnerable and more adversely impacted by the pandemic than others. This is an example of digital inequality, shown as the inequality in terms of access, usage, skills, and self-perceptions to digital engagement in individual and macro-level domains (60). Urgent attention and assistance should be given to these vulnerable people from policymakers and social welfare organizations.

Our study had some limitations. First, recall bias and social desirability bias could not be avoided in self-administered questionnaires. However, the use of communication methods and contents in family communication when the pandemic was severe was asked during the easing period of the pandemic, and recall errors would be little within such recent time periods. We used an online survey via emails without interviewers, which could help reduce social desirability bias (61). Second, although we tried to provide a clearer temporal sequence by asking the perceived outcomes during the easing period and the communication methods and contents during an earlier period when the pandemic was severe, due to the cross-sectional observational study design, we could not rule out reverse causality. Future prospective studies are needed to verify the associations and mediation effects we observed. Also, systematic bias due to residual confounding might exist. For example, people having face-to-face communication only could be lack of health literacy to share digital information with family, while sharing family life information through ICT tools were found to be associated with family wellbeing (17). Also, those having IM only might live separately with their family so face-to-face communication was unavailable. Such separation from family, especially amidst the pandemic, could lead to low family wellbeing or personal happiness. Third, as the COVID-19 pandemic changes rapidly and unpredictably, we tried to collect the largest possible sample within a short period, non-response bias could be present as younger and better-educated respondents were included. Generalization could be limited. Finally, details of the contents were not asked, and more in-depth information should be collected in future studies.

## Conclusions

We have first shown that, amidst the COVID-19 pandemic, better family wellbeing and personal happiness were associated with family communication using IM only and both methods than face-to-face only, and with positive and supportive contents. These associations were partially or almost fully mediated by communication quality. Family IM communication and positive and supportive contents may promote family wellbeing and personal happiness. People with no family communication may need urgent attention and assistance. Prospective studies are needed to verify the associations and mediations.

## Data Availability Statement

The dataset presented in this article is not readily available because the sharing of data to third parties was not mentioned in subjects' consent. Requests to access the dataset can be directed to the corresponding author.

## Ethics Statement

The studies involving human participants were reviewed and approved by Institutional Review Board of the University of Hong Kong/Hospital Authority Hong Kong West Cluster (Reference Number: UW 20-238). Informed consent was obtained from all participants included in this study.

## Author Contributions

WG and SS: formal analysis and writing—original draft. BW: data curation, project administration, and writing—review and editing. SW: methodology and writing—review and editing. AL: conceptualization and writing—review and editing. SH: conceptualization, methodology, and writing—review and editing. MW and TL: supervision, conceptualization, and writing—review and editing. All authors participated in the critical review of this study and provided final approval for publication submission.

## Funding

This study was funded by the Hong Kong Jockey Club Charities Trust.

## Conflict of Interest

The authors declare that the research was conducted in the absence of any commercial or financial relationships that could be construed as a potential conflict of interest.

## Publisher's Note

All claims expressed in this article are solely those of the authors and do not necessarily represent those of their affiliated organizations, or those of the publisher, the editors and the reviewers. Any product that may be evaluated in this article, or claim that may be made by its manufacturer, is not guaranteed or endorsed by the publisher.
