# Supplementary material for: Associations of Face-to-Face and Instant Messaging Family Communication and Their Contents With Family Wellbeing and Personal Happiness Amidst the COVID-19 Pandemic
Source: Front Psychiatry. 2022 Mar 29;13:780714. doi: 10.3389/fpsyt.2022.780714 (PMC9001841; doi:10.3389/fpsyt.2022.780714)
Supplement: Supplementary file 1 [file Table_1.DOCX]

Supplementary Material

**Supplementary Table 1**. Unweighted sociodemographic characteristics by communication methods (n=4891) ^a^

| Characteristics | No family communication (n=320, 6.5%) | | Face-to-face only (n=704, 14.4%) | | | IM only (n=1125, 23.0%) | | Both methods (n=2742, 56.1%) | Total  (n=4891) |
| --- | --- | --- | --- | --- | --- | --- | --- | --- | --- |
|  | n (%) | *P* (vs. both methods) ^c^ | n (%) | *P* (vs. both methods) ^c^ | n (%) | | *P* (vs. both methods) ^c^ | n (%) | n (%) |
| **Sex** |  | 0.02 |  | 0.07 |  | | <0.001 |  |  |
| Male | 166 (50.3) |  | 343 (48.7) |  | 397 (35.3) | |  | 1232 (44.9) | 2138 (43.7) |
| Female | 154 (49.7) |  | 361 (51.3) |  | 728 (64.7) | |  | 1510 (55.1) | 2753 (56.3) |
| **Age group (years)** |  | 0.29 |  | <0.001 |  | | <0.001 |  |  |
| 18-24 | 20 (6.3) |  | 50 (7.1) |  | 13 (1.2) | |  | 136 (5.0) | 219(4.5) |
| 25-44 | 169 (52.8) |  | 401 (57.0) |  | 531 (47.2) | |  | 1348 (49.1) | 2449 (50.1) |
| 45-64 | 121 (37.8) |  | 234 (33.2) |  | 516 (45.9) | |  | 1142 (41.7) | 2013 (41.2) |
| ≥65 | 10 (3.1) |  | 19 (2.7) |  | 65 (5.8) | |  | 116 (4.2) | 210 (4.43) |
| **Education** |  | 0.08 |  | <0.001 |  | | 0.71 |  |  |
| Secondary or below | 72 (10.3) |  | 72 (10.3) |  | 192 (17.2) | |  | 345 (12.7) | 659 (13.6) |
| Tertiary or above | 630 (89.7) |  | 630 (89.7) |  | 925 (82.8) | |  | 2381 (87.3) | 4199 (86.4) |
| **Monthly household income per person** | | 0.02 |  | <0.001 |  | | 0.48 |  |  |
| Lower | 96 (34.7) |  | 212 (35.6) |  | 289 (29.3) | |  | 673 (28.1) | 1270 (29.8) |
| Higher | 181 (65.3) |  | 383 (64.4) |  | 698 (70.7) | |  | 1724 (71.9) | 2986 (70.2) |
| **Housing type** |  | 0.02 |  | 0.06 |  | | 0.56 |  |  |
| Rented | 117 (37.9) |  | 282 (42.1) |  | 376 (34.4) | |  | 828 (31.2) | 1603 (33.9) |
| Owned | 192 (62.1) |  | 388 (57.9) |  | 716 (65.6) | |  | 1824 (68.8) | 3120 (66.1) |
| **Socioeconomic status** | | 0.02 |  | <0.001 |  | | 0.06 |  |  |
| Low | 64 (23.6) |  | 121 (21.0) |  | 196 (20.1) | |  | 409 (17.3) | 790 (18.9) |
| Medium | 96 (35.4) |  | 241 (41.9) |  | 347 (35.7) | |  | 813 (34.5) | 1497 (35.8) |
| High | 111 (41.0) |  | 213 (37.1) |  | 430 (44.2) | |  | 1137 (48.2) | 1891 (45.3) |
| **Number of cohabitants** |  | <0.001 |  | <0.001 |  | | <0.001 |  |  |
| Mean±SD | 1.7±1.4 |  | 2.3±1.2 |  | 1.9±1.3 | |  | 2.6±1.3 | 2.3±1.3 |

IM: Instant messaging. SD: Standard deviation.

^a^ Respondents with missing data were excluded. Total percentages may not be 100.0% after rounding. Frequencies may not add up to the total numbers after weighting.

^b^ Socioeconomic status: a composite score of education (0=secondary or below, 1=tertiary), income (0=lower, 1=higher), and housing (0=rented, 1=owned), analyzed as low (0-1), medium (2) and high (3).

^c^ Pairwise comparisons using Chi-square test for categorical variables and t-test for continuous variables with Bonferroni adjusted level of significance: 0.05/3=0.017.

**Supplementary Table 2**. Unweighted percentages of contents by communication methods ^a^

| Contents | Total | | |  | One method only | | | |  | | Both methods (n=2607) | | |
| --- | --- | --- | --- | --- | --- | --- | --- | --- | --- | --- | --- | --- | --- |
|  | Face-to-face, n (%) (n=3225) | IM messages, n (%) (n=3911) | *P* ^b^ |  | Face-to-face only, n (%) (n=619) | IM only, n (%) (n=1304) | *P* ^c^ |  | | n (%) | | *P* ^c^  (vs. face-to-face only | *P* ^c^  (vs. IM only) |
| Self/family-related things in daily life | 2783 (80.8) | 3196 (82.7) | 0.04 |  | 545 (77.4) | 961 (85.4) | <0.001 |  | | 2459 (89.7) | | <0.001 | <0.001 |
| Information of COVID-19 | 2772 (80.4) | 2349 (60.7) | <0.001 |  | 511 (72.6) | 622 (55.3) | <0.001 |  | | 2371 (86.5) | | <0.001 | <0.001 |
| Self/family-related happy/funny things | 1702 (49.4) | 1518 (39.3) | <0.001 |  | 263 (37.4) | 427 (38.0) | 0.80 |  | | 1607 (58.6) | | <0.001 | <0.001 |
| Others (e.g. daily life information, news, and current affairs, etc.) | 1629 (47.3) | 1547 (40.0) | <0.001 |  | 313 (44.5) | 421 (37.4) | <0.001 |  | | 1546 (56.4) | | <0.001 | <0.001 |
| Showing care | 1444 (41.9) | 1474 (38.1) | 0.001 |  | 216 (30.7) | 505 (44.9) | 0.03 |  | | 1414 (51.6) | | <0.001 | <0.001 |
| Other health information | 1089 (31.6) | 575 (14.9) | <0.001 |  | 172 (24.4) | 126 (11.2) | <0.001 |  | | 978 (35.7) | | <0.001 | <0.001 |
| Self/family-related unhappy things | 1030 (29.9) | 1357 (35.1) | <0.001 |  | 191 (27.1) | 430 (38.2) | <0.001 |  | | 1200 (43.8) | | <0.001 | 0.002 |
| Self/family-unrelated happy/funny things | 961 (27.9) | 703 (18.2) | <0.001 |  | 151 (21.5) | 159 (14.1) | <0.001 |  | | 949 (34.6) | | <0.001 | <0.001 |
| Encouragements | 530 (15.4) | 613 (15.9) | 0.58 |  | 62 (8.8) | 216 (19.2) | <0.001 |  | | 596 (21.7) | | <0.001 | 0.08 |
| Appreciations | 416 (12.1) | 321 (8.3) | <0.001 |  | 46 (6.5) | 105 (9.3) | <0.001 |  | | 440 (16.1) | | <0.001 | <0.001 |
| Good wishes | 338 (9.8) | 493 (12.8) | <0.001 |  | 33 (4.7) | 170 (15.1) | <0.001 |  | | 435 (15.9) | | <0.001 | 0.56 |
| **Different kinds of contents** ^d^ | |  |  |  |  |  |  |  | |  | |  |  |
| Neutral contents | 3391 (98.4) | 3343 (86.4) | <0.001 |  | 688 (97.7) | 949 (84.4) | <0.001 |  | | 2726 (99.4) | | <0.001 | <0.001 |
| Positive contents | 1883 (54.6) | 1739 (45.0) | <0.001 |  | 313 (44.5) | 496 (44.1) | 0.88 |  | | 1748 (63.8) | | <0.001 | <0.001 |
| Supportive contents | 1543 (44.8) | 1646 (42.6) | 0.06 |  | 230 (32.7) | 566 (50.3) | <0.001 |  | | 1509 (55.0) | | <0.001 | 0.01 |
| Negative contents | 1089 (31.6) | 575 (14.9) | <0.001 |  | 172 (24.4) | 126 (11.2) | <0.001 |  | | 978 (35.7) | | <0.001 | <0.001 |

IM: Instant messaging.

^a^ Weighted by sex, age, and education of the 2019 Hong Kong census data. Respondents with no family communication and those with missing data were excluded. Contents were ranked by their weighted percentages in total face-to-face.

^b^ Chi-square test with level of significance=0.05.

^c^ Pairwise comparisons using Chi-square test and Bonferroni adjusted level of significance: 0.05/3=0.017.

^d^ Neutral contents: self/family-related things in daily life, information of COVID-19, other health information, and others (e.g. daily life information, news, and current affairs, etc.). Positive contents: self/family-related and unrelated happy/funny things. Supportive contents: showing care, encouragements, appreciations, and good wishes. Negative contents: self/family-related unhappy things.
